# Value of flaccid penile ultrasound in screening for arteriogenic impotence: a preliminary prospective study

**DOI:** 10.1186/s12880-018-0284-2

**Published:** 2018-11-06

**Authors:** Li-Da Chen, Fu-Shun Pan, Lu-Yao Zhou, Yu-Bo Liu, Jian-Yao Lv, Ming Xu, Xiao-Yan Xie, Ming-De Lu, Zhu Wang, Wei Wang

**Affiliations:** 1 Department of Medical Ultrasonics, Institute of Diagnostic and Interventional Ultrasound, The First Affiliated Hospital of Sun Yat-Sen University, 58 Zhongshan Road 2, Guangzhou, 510080 People’s Republic of China; 2 Department of Ultrasound, State Key Laboratory of Oncology in South China, Sun Yat-Sen University Cancer Center, Guangzhou, China; 3 Department of Hepatobiliary Surgery, The First Affiliated Hospital of Sun Yat-Sen University, Guangzhou, China

**Keywords:** Erectile dysfunction, Ultrasound, Peak systolic velocity, Receiver operating characteristic, International index of erectile function, Erectile hardness grading scale

## Abstract

**Background:**

This prospective study is to evaluate the potential value of sonographic measurements in the flaccid penis for the screening of arteriogenic impotence.

**Methods:**

A consecutive series of 260 Chinese males consulting for sexual dysfunction and 54 controls underwent sonographic examination. The sonographic parameters were correlated with the clinical gold standards, including the international index of erectile function (IIEF) and penile erectile hardness grading scale (EHGS). The sensitivity, specificity, positive predictive value (PPV), negative predictive value (NPV) and area under the receiver operating characteristic curve (AUROC) of flaccid peak systolic velocity (PSV) in predicting patients with normal function were analyzed.

**Results:**

The mean cavernous PSV of both sides in the patients with sexual dysfunction ranged from 7.76 to 11.12 cm/sec with a stepwise increase in IIEF and EHGS grading scale (*P* < .05). The cutoff value of flaccid PSV for the differential diagnosis of grade 4 of IIEF-5 or EHGS was 8.20–8.90 cm/sec, with an AUROC of 0.657–0.724, specificity of 82.96–86.84% and PPV of 95.20–96.60%, respectively.

**Conclusions:**

This simple flaccid PSV measurement is a specific tool for screening arteriogenic impotence.

## Background

Erectile dysfunction (ED) is a common worldwide and potentially treatable problem with an incidence of 50% in the general male population aged between 40 and 70 years [1]. In addition to psychological and metabolic factors and relational problems, arteriogenic causes play important roles in erectile dysfunction [2–4]. Changes in any one of these factors may result in ED.

For diagnosis of arteriogenic causes, color Doppler ultrasound is one of the most noninvasive, simple and promising tools [1–3, 5–8]. The peak systolic velocity (PSV) of the cavernous artery measured after the intra-cavernous injection (ICI) of vasoactive agents is a widely accepted criterion for evaluating penile circulation [2, 6]. A post-ICI PSV value less than 25 cm/sec is recognized as a severely insufficient arterial supply [2, 5]. However, the clinical adaptability of this method is relatively limited because of the time consuming, lack of standardization, and side effects due to vasoactive agents [2]. Currently, a growing number of male are anxious about their sexual lives. A simple parameter for evaluating penile arteriogenic health, with limited costs in terms of time and money and without the inconvenience of priapism, is an ideal goal. Therefore, post-ICI PSV is not an optimal method of screening for arteriogenic impotence.

Doppler investigation in the flaccid state would avoid these disadvantages. It has been reported that PSV values that were measured in the flaccid state show a significant correlation with post-ICI PSV and might be predictive in the determination of arterial insufficiency [2, 7, 9]. However, these studies all used the PSV measured in the flaccid state as an additional tool to evaluate penile circulation. In our opinion, the evaluation of PSV in the flaccid penis alone for screening arteriogenic impotence remains underestimated. It has been reported that flaccid PSV in the general population is above 13 cm/sec for Europeans [2], but no data about Chinese population has been reported.

In this prospective study, we tried to evaluate the potential value of ultrasound (US) parameters measured in the flaccid state for the diagnosis of arteriogenic impotence, correlated with the international index of erectile function (IIEF) and penile erectile hardness grading scale (EHGS) [10–12].

## Methods

### Patients population

This prospective study was approved by the research ethics board of our institution, and informed consent was obtained. Participants recruited from our hospital consented to receive this noninvasive US examination, and this aspect of the study was approved by the Ethical Committee of the First Affiliated Hospital of Sun Yat-Sen University as IRB_2011 [168] entitled “Assessment of cavernous endothelial dysfunction in patients consulting for sexual dysfunction”. From October 2014 to October 2016, a consecutive series of 310 Chinese male patients consulting for sexual dysfunction were referred. Among them, 6 patients declined the US examination. Another 44 patients were excluded if (a) US data were not collected according to the standard protocol (*n* = 25), (b) no reference standard was obtained (*n* = 13), or (c) clinical or US data were missing (*n* = 6).The remaining 260 eligible patients underwent color Doppler sonographic examination. Fifty-four healthy adult volunteers who did not have a history of sexual dysfunction were examined by US and served as a control group for which the same US parameters were obtained. No volunteers had taken any medication or drugs at the time of the US examination. The mean ages of the two groups were 32.9 ± 8.3 years (range 19–72 years) and 29.9 ± 8.9 years (range 19–40 years), respectively (*P* = 0.200). All patients were in a stable monogamous relationship with a female partner and had made at least one attempt at sexual intercourse over the last 8 weeks. All patients underwent an erectile dysfunction evaluation that included IIEF-5 and EHGS.

### US examination

All patients in each group were examined using an Aplio XV or 500 (Toshiba Medical Systems, Tokyo, Japan) or Mylab Twice (Esaote Medical Systems, Genoa, Italy) by three operators (W.W., Z. W., L.Y Z.). To ensure patient privacy, all exams were performed in a quiet, comfortable room. Excessive compression with the transducer was avoided. First, in grayscale US, the penis was evaluated in both the longitudinal and transverse planes in the flaccid state. Then, color Doppler sonography was optimized to obtain the best longitudinal plane of the cavernosal arteries. In this longitudinal plane, spectral analysis of the cavernosal arteries was performed in the proximal part of the penis. The optimal site for spectral analysis was the proximal part of the cavernosal arteries where the vessels curved. This location allowed an angle of insonation as low as < 30° for accurate angle-corrected velocity calculations. The PSV, resistance index (RI) and diameters of both cavernosal arteries were recorded. When analyzing the spectral Doppler, the optimized pulse repetition frequency and wall filter were selected, and the width of the Doppler sample size was set at 0.5 mm - 1 mm. Three consecutive similar waveforms were considered to constitute a satisfactory test.

### IIEF-5 and EHGS

The IIEF-5, a 5-item questionnaire, is used for clinical diagnosis of the severity of ED, including scores on the 5-item form (that is, Erection confidence, Penetration ability, Maintenance frequency, Maintenance ability, Intercourse satisfaction) [12]. These items focus on erectile function and intercourse satisfaction. This tool has become the ‘gold standard’ for the clinical evaluation of therapy efficacy. The degree of ED is classified as follows: grade1 = severe ED (scores between 5 and 7), grade2 = mild ED (scores between 8 and 11), grade 3 = moderate ED (scores between 12 and 21), grade 4 = no ED (scores between 22 and 25).

The erection hardness grading scale (EHGS) was developed in 1998 by Goldstein et al. [11]. It is a convenient, four-grade scale for ED that provides a reliable measure of the degree and duration of penile rigidity, according to data reported at the European Association of Urology. The erection hardness of the penis is graded according to the EHGS as follows: grade 1 = increased in size without hardness, grade 2 = hard but not hard enough for penetration, grade 3 = hard enough for penetration but not completely hard, and grade 4 = completely hard and fully rigid.

### Statistical analysis

The data were expressed as the mean ± SD or median and inter-quartile range (IQR), as appropriate. The chi-square test or Fisher’s exact test was used to evaluate the difference between the IIEF-5 and EHGS groups in PSV, diameter and RI. Receiver operating characteristic (ROC) curves were compared to evaluate the diagnostic performance of PSV using the MedCalc version 9.0 software (MedCalc Software, Mariakerke, Belgium). The diagnostic performance was expressed as the area under the ROC curve (AUROC). The sensitivity, specificity, accuracy, positive predictive value (PPV) and negative predictive value (NPV) were calculated. Figures were drawn using the Origin 8.5 software (OriginLab, Northampton, MA, USA). *P* < 0.05 was considered to indicate statistical significance.

## Results

### Relationships between US parameters and IIEF-5 and EHGS

**Fig. 1:**
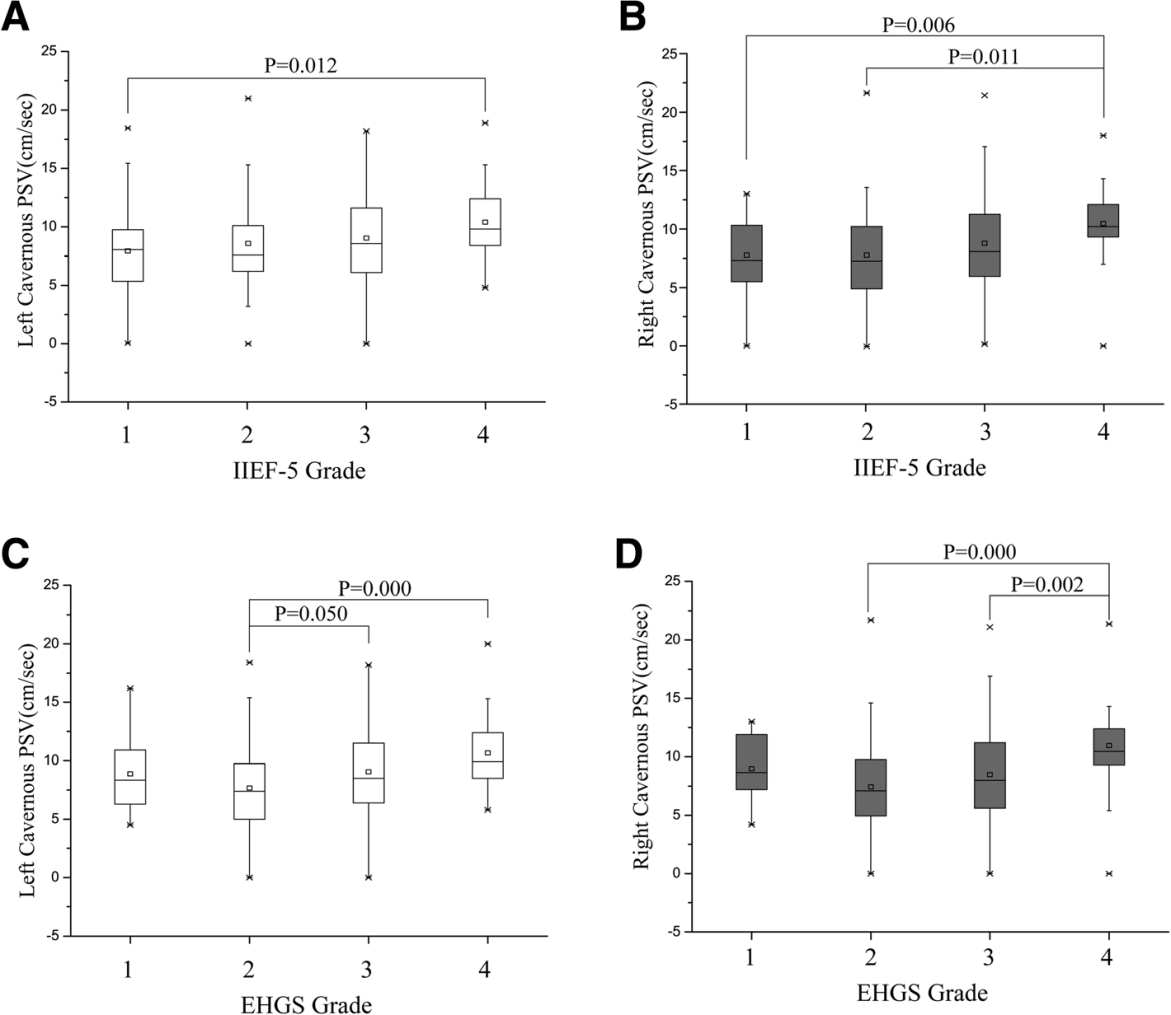
Box plots show the range between the 25^th^and 75th percentiles (box), mean (small square in the box), median (horizontal line in the box), and outliers (whiskers) of flaccid cavernous PSV. A stepwise increase in both flaccid PSV values is observed with increasing grades of IIEF-5 or EHGS (*P* < 0.05)

The IIEF-5 and EHGS of ED patients are shown in Table 1. The mean PSVs of both sides in the patients consulting for sexual dysfunction ranged from 7.76 to 11.12 cm/sec with a stepwise increase in IIEF and EHGS grading (*P* < .05) (Table 1, Fig. 1).Moreover, the differences between grade 4 of IIEF-5 or EHGS and other grades were significant (grade 1 vs. 4 in the left cavernous PSV, grade1 or 2 vs. 4 in the right cavernous PSV for IIEF-5; grade 2 vs. 4 in the left cavernous PSV, grade 2 or 3 vs. 4 in the right cavernous PSV for EHGS, all *P* < .05) (Fig. 1).

**Table 1:** IIEF-5 and EHGS findings, Cavernous PSV, Diameter and RI Measurements in Patients with ED

Parameters	No. of Patients	Left Cavernous Artery							Right Cavernous Artery						
		PSV(cm/s)		Diameter(mm)		RI			PSV(cm/s)		Diameter(mm)		RI		
		Mean ± SD	P* Value	Mean ± SD	P* Value	Median(Mean ± SD)		P* Value	Mean ± SD	P* Value	Mean ± SD	P* Value	Median(Mean ± SD)		P* Value
IIEF-5			0.025		0.142			0.528		0.012		0.307			0.174
1	57	7.87 ± 3.44		0.50 ± 0.16		1.00	0.93 ± 0.20		7.76 ± 3.18		0.49 ± 0.17		1.00	0.93 ± 0.20	
2	48	8.56 ± 4.14		0.49 ± 0.16		1.00	0.92 ± 0.22		7.81 ± 4.01		0.46 ± 0.18		1.00	0.92 ± 0.22	
3	135	8.86 ± 3.75		0.53 ± 0.16		1.00	0.97 ± 0.13		8.46 ± 3.97		0.50 ± 0.16		1.00	0.96 ± 0.16	
4	20	10.82 ± 3.29		0.58 ± 0.18		1.00	0.97 ± 0.08		10.82 ± 2.79		0.54 ± 0.15		1.00	0.99 ± 0.03	
EHGS			0.004		0.172			0.073		0.001		0.093			0.197
1	11	8.68 ± 3.53		0.58 ± 0.13		1.00	0.99 ± 0.04		8.85 ± 2.75		0.59 ± 0.19		1.00	1.00 ± 0.00	
2	79	7.70 ± 3.58		0.49 ± 0.16		1.00	0.91 ± 0.23		7.43 ± 3.54		0.48 ± 0.17		1.00	0.92 ± 0.23	
3	148	9.00 ± 3.88		0.52 ± 0.16		1.00	0.97 ± 0.13		8.42 ± 3.90		0.49 ± 0.16		1.00	0.96 ± 0.16	
4	22	10.79 ± 2.84		0.56 ± 0.18		1.00	0.96 ± 0.08		11.12 ± 3.20		0.54 ± 0.13		1.00	0.99 ± 0.04	

*Comparisons among IIEF-5 subgroups or EHGS subgroups were made by using the variance analysis

Diameter and RI in both sides of the cavernous artery showed no significant difference among different grades of IIEF-5 or EHGS (all *P* > .05), with a mean value of 0.48–0.58 mm for diameter and a median value of 1.00 for RI.

**Fig. 2:**
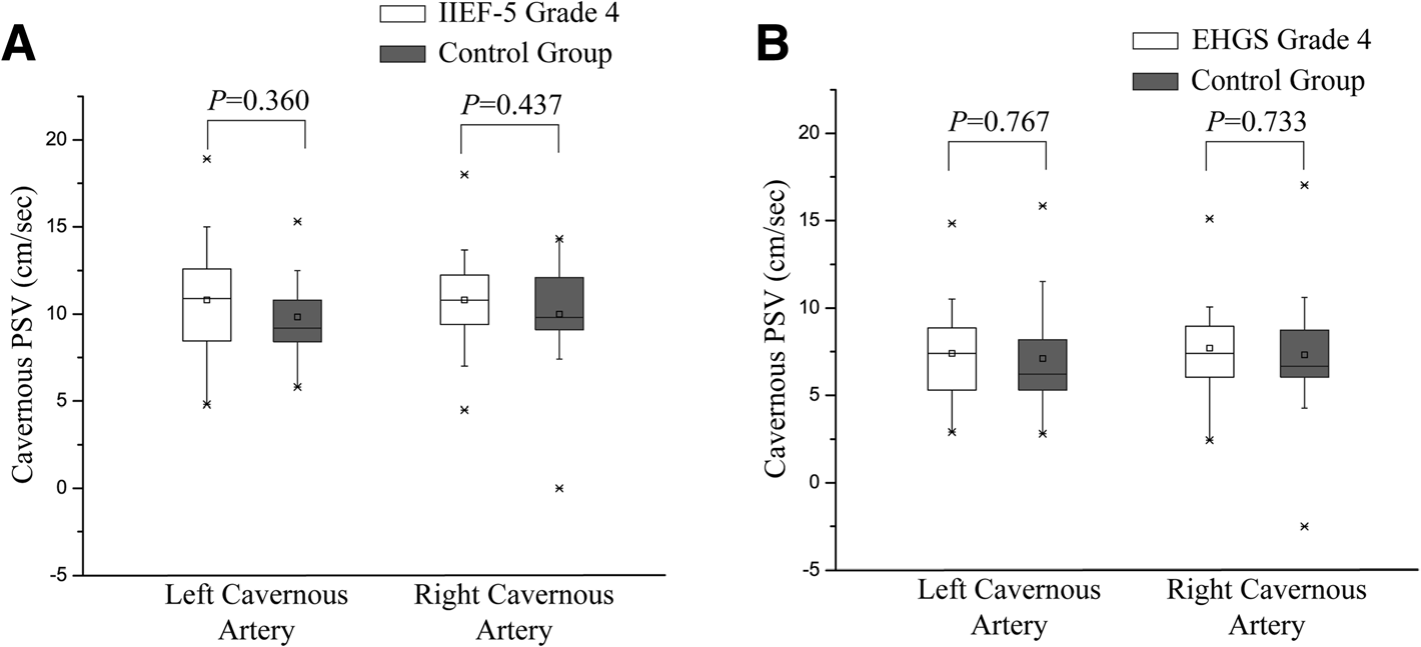
Box plots show the range between the 25th and 75th percentiles (box), mean (small square in the box), median (horizontal line in the box), and outliers (whiskers) of flaccid cavernous PSV. PSV in the flaccid state in patients consulting for sexual dysfunctions with grade 4 of IIEF-5 or EHGS show no statistically significant difference from the control group (*P* > .05)

The flaccid PSV on both sides in the patients consulting for sexual dysfunctions with grade 4 of IIEF-5 or EHGS showed no statistically significant difference from the control group (*P* > .05) (Fig. 2).In the control group, 45/54 (83.3%) volunteers were classified as grade 4 of IIEF-5, and 48/54 (88.9%) were classified as grade 4 of EHGS.

### Receiver operating characteristic curves

**Fig. 3:**
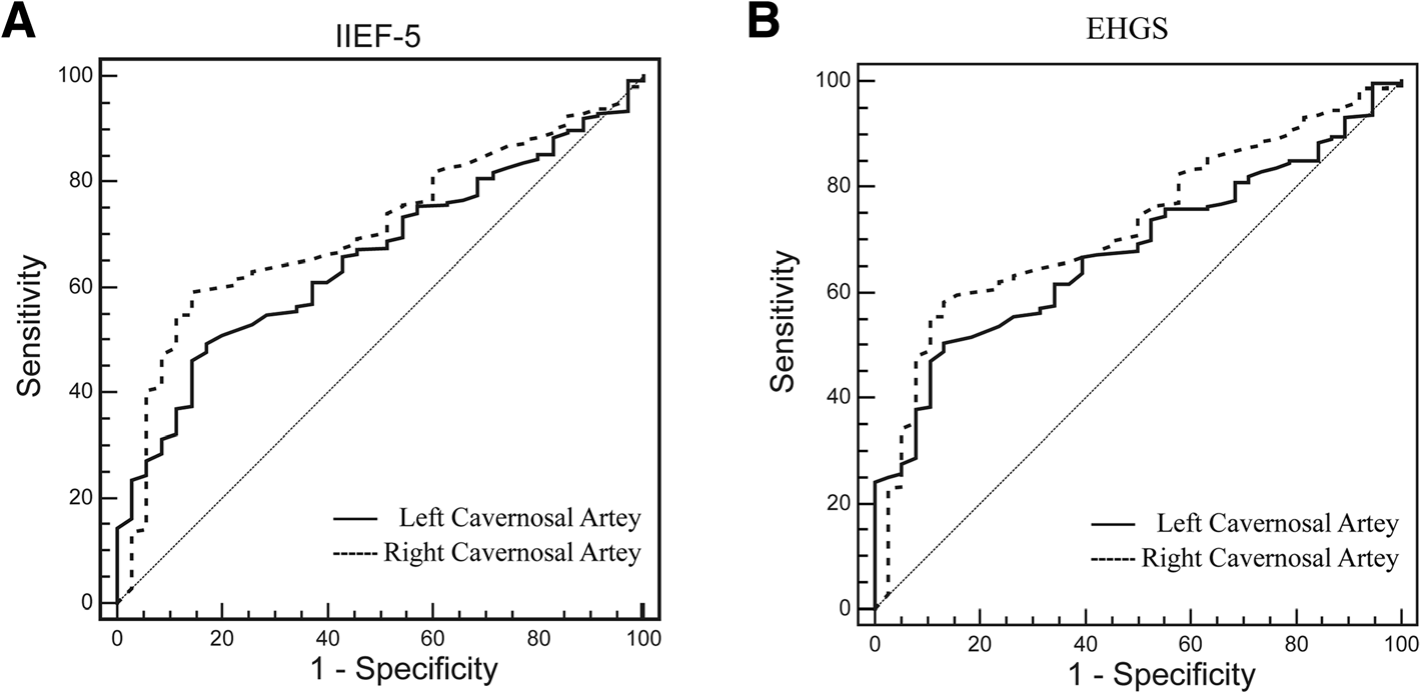
Receiver operating characteristic curves for estimation of the performance of flaccid PSV in patients with grade 4 of IIEF-5 or EHGS

The AUROC for estimating the performance of flaccid PSV in the patients with no ED (grade 4 of IIEF-5, scores between 22 and 25) was 0.657 (cutoff value, 8.20 m/sec; sensitivity, 49.38%; specificity, 82.86%; PPV, 95.20%; NPV, 19.10%) for the left cavernous artery and 0.706 (cutoff value, 8.90 m/sec; sensitivity, 59.26%; specificity, 85.71%; PPV, 96.60%; NPV, 23.30%) for the right cavernous artery (Fig. 3, Table 2).

**Table 2:** Cavernous PSV Measurements for Determination of ED

Parameter	IIEF-5 Grade 1–3 vs. 4		EHGS Grade 1–3 vs. 4	
	Left Cavernous Artery	Right Cavernous Artery	Left Cavernous Artery	Right Cavernous Artery
Cutoff Value (cm/sec)	8.20	8.90	8.20	8.70
AUC	0.657	0.706	0.679	0.724
95% Confidence Interval	0.598 to 0.713	0.649 to 0.759	0.620 to 0.733	0.668 to 0.776
Sensitivity (%)	49.38	59.26	50.42	58.33
Specificity (%)	82.86	85.71	86.84	86.84
Positive Predictive Value (%)	95.20	96.60	96.00	96.60
Negative Predictive Value (%)	19.10	23.30	21.70	24.80

The AUROC for estimating the performance of flaccid PSV in the patients with grade 4 of EHGS (completely hard and fully rigid) was 0.679 (cutoff value, 8.20 m/sec; sensitivity, 50.42%; specificity, 86.84%; PPV, 96.00%; NPV, 21.70%) for the left cavernous artery and 0.724 (cutoff value, 8.70 m/sec; sensitivity, 58.33%; specificity, 86.84%; PPV, 96.60%; NPV, 24.80%) for the left cavernous artery (Fig. 3, Table 2).

## Discussion

This study was performed to determine the potential value of sonographic measurements in the flaccid penis for diagnosing arteriogenic impotence. Our prospective study demonstrated that flaccid PSV values were correlated with IIEF-5 and EHGS grading. The optimal cutoff for PSV is 8.2–8.9 cm/sec, which yielded a specificity and PPV of approximately 82–87 and 95%–97%, respectively.

The incidence of ED in adults is rapidly increasing [13]. Although there are complicating factors, arteriogenic impotence is among the most important cause of erectile dysfunction [14–16]. Color Doppler sonographic examination is a noteworthy diagnostic tool for detecting failure of the penile arterial supply [2, 5]. For patients consulting for sexual dysfunction, the most commonly used evaluation is Doppler investigation in conjunction with ICI of vasoactive substances. The proposed cutoff values of post-ICI PSV for arterial insufficiency range widely, from 25 to 40 cm/sec [2]. Meanwhile, Doppler investigation after ICI is a time-consuming technique. Psychological inhibition and anxiety during ICI may disturb the assessment of arterial supply [1].

Kahvecioglu et al. [7] had demonstrated that flow in the cavernosal arteries in the flaccid state could determine nondiabetic patients with vasculogenic impotence with a high accuracy rate. Furthermore, Corona et al. [2] found that flaccid PSV showed a significant (*r* = 0.513, *P* < 0.0001) correlation with post-ICI PSV, similar to that (*r* = 0.477) proposed by Mancini et al. [17]. They also concluded that flaccid PSV < 13 cm/s predicted reduced dynamic PSV with an accuracy greater than 80%, the cut off of which was higher than that previously reported by Sen et al. and Roy et al. [5, 6]. Those European reports had documented normal flaccid PSV values varied from 10 cm/s to 25 cm/s, but these results were not applicable for Asians. Furthermore, the utility of flaccid PSV alone had not been sufficiently studied. In our study, we aimed to test this easier method’s correlation with the clinical gold standard. The correlations between flow parameters and IIEF-5 or EHGS were evaluated. Only the PSV in both sides showed significant differences among the different IIEF-5 or EHGS groups. Furthermore, there was no difference between grade 4 of IIEF-5 or EHGS and the control group (all *P* > 0.05), which may indicate that for most patients consulting for sexual dysfunction in grade 4, arteriogenic impotence is a minimal factor.

Considering the patients consulting for sexual dysfunction and the controls as a whole, the ROC curve indicated that a threshold of 8.2–8.9 cm/sec should be chosen. The AUROC of PSV diagnosis was 0.657–0.724, the sensitivity was as low as 49.38%, the specificity was high at 82.96–86.84%, and the PPV values were 95.2–96.6%. The findings of the current study indicated that when the PSV cutoff was set at 8.2–8.9 cm/sec, it was possible to distinguish arteriogenic causes from psychological factors. We believe that this finding is of interest because a PSV of 8.2 cm/sec or lower could be recognized as associated with a higher risk of arteriogenic incompetence in the Chinese population. In this study, our data showed that the flaccid penile US evaluation has the potential to become a specific tool to diagnose arteriogenic impotence. Nonetheless, many other factors regulate sexual function in ED patients, which may explain the low sensitivity and NPV in this study.

One limitation of our study is that we did not include post-ICI PSV as a reference standard because of the complexity and invasiveness. We intended to explore the clinical importance of flaccid PSV for screening for arteriogenic impotence. It was suggested for high-risk patients as the first-line exam at the high PPV of 96%. However, flaccid penile Doppler is not sufficient for management decision making at a low sensitivity of 50 to 60%. Moreover, It has been reported that up to 47% of patients with a diagnosis of venous leakage by dynamic infusion cavernosometry showed completely normal hemodynamics [18, 19]. Repeated US exam with ICI was mandatory to exclude venous leakage [20]. Second, the mean age of the patients in our study was lower than the general mean age of patients with ED, which might be due to selection bias. The percentage of patients between 20 years and 39 years in our study is 80.8% (210/260). We infer that those men are in an active stage of their sexual life with higher expectations. Thus, they were more likely to seek help from doctors when they were included in our study.

## Conclusion

In conclusion, our results show that flaccid PSV values correlate with IIEF-5 or EHGS grading in the Chinese population. The best flaccid PSV cutoff value was 8.2–8.9 cm/sec, with a specificity of 82.96–86.84% and PPV of 95.2–96.6%. This easily performed method has the potential to become a specific tool in screening for arteriogenic impotence.

## Abbreviations

AUROC: Area under the receiver operating characteristic curve
ED: Erectile dysfunction
EHGS: Penile erectile hardness grading scale
ICI: Intra-cavernous injection
IIEF: International index of erectile function
IQR: Inter-quartile range
NPV: Negative predictive value
PPV: Positive predictive value
PSV: Peak systolic velocity
RI: Resistance index
ROC: Receiver operating characteristic
US: Ultrasound
